# Splenectomy increases the survival time of heart allograft via developing immune tolerance

**DOI:** 10.1186/1749-8090-8-129

**Published:** 2013-05-16

**Authors:** Jinguo Zhu, Shuzhen Chen, Jinju Wang, Cheng Zhang, Wei Zhang, Peng Liu, Ruilian Ma, Yanfang Chen, Zhen Yao

**Affiliations:** 1 Department of Cardiothoracic Surgery, The People’s Hospital of Sanya, Sanya, Hainan, 572000, China; 2 Department of Cardiology, The People’s Hospital of Sanya, Sanya, Hainan, 572000, China; 3 Department of Pharmacology, The People’s Hospital of Sanya, Sanya, Hainan, 572000, China; 4 Department of Pharmacology and Toxicology, Boonshoft School of Medicine of Wright State University, Dayton, OH, 45435, USA

**Keywords:** Splenectomy, Heart transplantation, Immune tolerance, CD_4_^+^CD_25_^+^ Treg, Lymphocyte apoptosis

## Abstract

**Background:**

The spleen is an active lymphoid organ. The effect of splenectomy on the immune response remains unclear. This study investigated whether splenectomy can induce immune tolerance and has a beneficial role in cardiac allograft.

**Methods:**

Wistar rats were used for heart donors. The Sprague–Dawley (SD) rats designated as the recipients of heart transplantation (HT) were randomly assigned into four groups: sham, splenectomy, HT, splenectomy + HT. The survival of transplanted hearts was assessed by daily checking of abdominal palpation. At various time points after transplantation, the transplanted hearts were collected and histologically examined; the level of CD_4_^+^CD_25_^+^ T regulatory lymphocytes (Tregs) and rate of lymphocyte apoptosis (annexin-v^+^ PI^+^ cells) in the blood were analyzed by using flow cytometric method.

**Results:**

1) Splenectomy significantly prolonged the mean survival time of heart allografts (7 ± 1.1 days and 27 ± 1.5 days for HT and splenectomy + HT, respectively; n = 12-14/group, HT *vs.* splenectomy + HT, p < 0.001); 2) Splenectomy delayed pathological changes (inflammatory cell infiltration, myocardial damage) of the transplanted hearts in splenectomy + HT rats; 3) The level of CD_4_^+^CD_25_^+^ Tregs in the blood of splenectomized rats was significantly increased within 7 days (2.4 ± 0.5%*,* 4.9 ± 1.3% and 5.3 ± 1.0% for sham, splenectomy and splenectomy + HT, respectively; n = 15/group, sham *vs.* splenectomy or splenectomy + HT, p < 0.05) after splenectomy surgery and gradually decreased to baseline level; 4) Splenectomy increased the rate of lymphocyte apoptosis (day 7: 0.3 ± 0.05%, 3.9 ± 0.9% and 4.1 ± 0.9% for sham, splenectomy and splenectomy + HT, respectively; n = 15/group, sham *vs.* splenectomy or splenectomy + HT, p < 0.05) in a pattern similar to the change of the CD_4_^+^CD_25_^+^ Tregs in the blood.

**Conclusions:**

Splenectomy inhibits the development of pathology and prolongs the survival time of cardiac allograft. The responsible mechanism is associated with induction of immune tolerance via elevating CD_4_^+^CD_25_^+^ Tregs and increasing lymphocyte apoptosis.

## Background

Cardiac transplantation is the preferred surgical therapy for patients with end-stage heart diseases. However, allograft rejection is the major challenge for the transplantation [1]. To date, several strategies have been made towards preservation of the transplanted heart by regulating the immune response of the recipients. These include administration of immunosuppressive drugs [2] and generation of tolerogenic dendritic cells [3]. Nevertheless, the obstacle such as the adverse effects jeopardizes the clinical application [4]. Therefore, new avenues are demanded for successful cardiac transplantation.

Transplant immune tolerance is defined as a lack of transplant rejection in the recipient in the absence of immunosuppressive agents. CD4^+^CD25^+^ Tregs are a minor subset (5 - 10%) of CD4^+^ T cells, arising in the thymus. Accumulating data showed that the CD4^+^CD25^+^ Tregs potentially contribute to the development of transplant immune tolerance. For example, Sakaguchi and colleagues reported that removal of CD4^+^CD25^+^ Tregs from normal mice reduced the survival of the grafts [5]. Graca *et al.*[6] demonstrated that CD4^+^CD25^+^ Tregs are the principle regulator of transplantation tolerance by suppressing the active T cells.

The spleen is the largest single lymphoid organ in the body. It plays a critical role in the immune response. Several studies have investigated the effects of splenectomy on the immune system. Thomas et al. [7] showed that splenectomy prevented the hyperacute rejection of rabbit renal allograft. Carobbi and colleagues [8] found that splenectomy increased the survival rate of cardiac xenograft by blocking the humoral antibody response. However, the effect of splenectomy on immune tolerance of cardiac transplantation remains unclear.

The objective of this study was to determine whether splenectomy can increase the survive time of heart allograft by developing immune tolerance via augmenting Tregs and increasing lymphocyte apoptosis.

## Methods

### Animals

Adult Wistar and Sprague–Dawley (SD) rats (weight, 250 - 300 g) were used as donors and recipients, respectively. All rats were purchased from the Medical Experimental Animal Center of Sun Yat-sen University and housed in the animal care facility at Sun Yat-sen University. All rats were kept under standard temperature, humidity, and time light conditions and fed standard chow and water ad libitum. All experimental protocols were reviewed and approved by the Institutional Animal Care and Use Committee of Sun Yat-sen University.

### Experiment groups

All SD rats were randomly assigned to four groups (n = 15/group): sham; splenectomy; HT; splenectomy + HT.

### Splenectomy and heterotopic heart transplantation

In the sham group, the SD rats underwent laparotomy only. In splenectomy and splenectomy + HT groups, the splenectomy surgery was performed according to a previous published method [9]. Briefly, the rats were anaesthetized by a single intraperitoneal injection of ketamine/xylazine (100:10 μg/kg). After the spleen was surgically exposed and Hilar vessels were clamped, the spleen was removed and the vessel stump was ligated with suture. In HT and splenectomy + HT groups, hearts from Wistar rats (donors) were transplanted into SD rats (recipients) as previously reported with slight modifications [10-12]. In brief, the SD rats were anaesthetized as above described. A midline incision was made on the abdominal wall. By using standard vascular microsurgical techniques, the recipient’s abdominal aorta was anastomosed end-to-side to the donor’s abdominal aorta and the recipient’s inferior vena cava was anastomosed to the donor’s pulmonary artery. Graft survival was monitored by daily checking of palpable heartbeat. Graft rejection was defined as the cessation of heartbeat.

### Isolation of mononuclear cells from peripheral blood

A volume of 0.3-0.5 ml of peripheral blood was taken into a sterile heparinized syringe from the caudal vein of the SD rats in sham, splenectomy, and splenectomy + HT groups at various time points (day 1, 3, 5, 7, 15, 20, and 28) after sham or splenectomy surgery, or from the SD rats in HT group at day 1, 3, 5, and 7 after HT transplantation surgery. The peripheral blood mononuclear cells (PBMCs) were prepared by gradient density centrifugation as we described previously with slight modifications [13]. Briefly, blood was diluted in 2 ml PBS and then gently layered over equal volume of gradient medium (Hisopaque-1083, Sigma, Fairfax, VA) for centrifugation at 800 g for 30 mins at 4°C. The PBMCs in the interface layer were transferred to a new tube, washed with PBS, centrifuged at 400 g for 5 mins at 4°C and used for flow cytometric analysis.

### Analysis of CD4^+^CD25^+^ Tregs by flow cytometry

The Tregs were identified by double positive expression of membrane specific markers CD4 and CD25. The percentage of CD4^+^CD25^+^ Tregs in the peripheral blood from all experiment groups was analyzed at different time points (day 1, 3, 5, 7, 15, 20, and 28). The PBMCs were collected as described above and resuspended with PBS and incubated with FITC-conjugated anti-rat CD4 (eBioscience, San Diego, CA) and PE-conjugated anti-rat CD25 (eBioscience, San Diego, CA) antibodies for 30 mins at 4°C in the dark. Isotype matched (IgG) non-specific antibodies served as negative controls. The concentrations of antibodies were applied according to manufacture instructions. A total of at least 10 000 events were collected and analyzed by using Accuri C6 flow cytometer and CFlow Plus Analysis software (Ann Arbor, MI). A live lymphocyte gate was created on dot plots using forward scatter and side scatter plots. The percentage of Tregs was determined within the settled gate by double positive staining of CD4 and CD25.

### Analysis of lymphocyte apoptosis by flow cytometry

At different time points (day 1, 3, 5, 7, 15, 20, and 28), the PBMCs were collected from all experimental groups as described above to assess lymphocyte apoptosis. Apoptosis was measured by detecting phosphatidylserine externalization in the cell membrane using the annexin-v/propidium iodide (PI) assay. In brief, the collected cells were washed twice with cold PBS, centrifuged, resuspended in 100 μl of binding buffer containing 5 μl FITC-conjugated annexin-v (Invitrogen, Carlsbad, CA) and 2 μl 100 μg/ml PI (Invitrogen, Carlsbad, CA) and incubated for 15 mins at room temperature in the dark. Isotype matched (IgG) non-specific antibodies served as negative controls. The concentrations of antibodies were applied according to manufacture instructions. A total of at least 10 000 events were collected and analyzed by using Accuri C6 flow cytometer and CFlow Plus Analysis software (Ann Arbor, MI). A live lymphocyte gate was created on dot plots using forward scatter and side scatter plots. The rate of lymphocyte apoptosis was determined within the settled gate by double positive staining of annexin-v and PI.

### Histological examination of heart

The heart samples were collected from either splenectomy group or splenectomy + HT group. The heart samples harvested from donor rats served as the control group. All tissue samples were fixed in 4% buffered formalin solution overnight, embedded in paraffin, sectioned (5–6 μm thickness) under a microtome, and stained with hematoxylin and eosin (H&E) by using the standard method. All samples were analyzed under light microscopy in a blinded fashion.

### Statistical analysis

Data were expressed as mean ± SEM. Means for two groups were compared using Student’s *t*-test. Multiple comparisons were performed by one-way ANOVA. Graft survival was plotted using Kaplan-Meier method, and allograft survival rates were analyzed by using the log-rank test. P values <0.05 were considered statistically significant.

## Results

### Splenectomy prolongs the mean survival time of heart allografts

**Figure 1:**
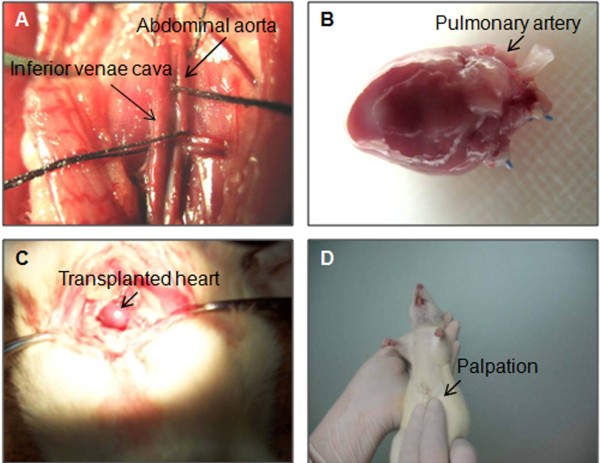
**Heterotopic heart transplantation surgery.** Representative images show: **A**, the abdominal aorta and inferior vena cava of a SD rat; **B**, the heart ready for transplantation; **C**, a SD rat with a transplanted heart; **D**, detecting heartbeat of transplanted heart by palpation.

**Figure 2:**
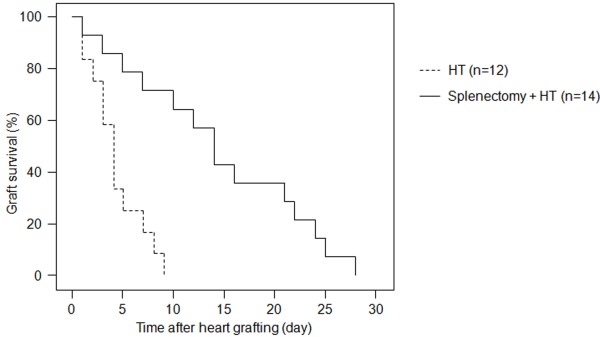
**Assessment the mean survival time of cardiac allograft.** The Kaplan–Meier curve shows the survival time of transplanted hearts. The mean allograft survival time was significantly prolonged in splenectomy + HT group (—) when compared with cardiac allograft alone rats in HT group (− −) (7 ± 1.1 days and 27 ± 1.5 days for HT and splenectomy + HT, n = 12-14/group, HT *vs.* splenectomy + HT, p < 0.001).

The representative images of heart transplantation are presented in Figure 1. The survival time of transplanted hearts in HT group was 7 ± 1.1 days (n = 12), while the survival time of transplanted hearts in splenectomy + HT group was 27 ± 1.5 days (n = 14). The data showed that the mean survival time of heart allograft in splenectomized rats was significantly longer (*P* < 0.001) than that in non-splenectomy rats (Figure 2).

### The level of CD4^+^ CD25^+^ Tregs was increased in splenectomized rats

**Figure 3:**
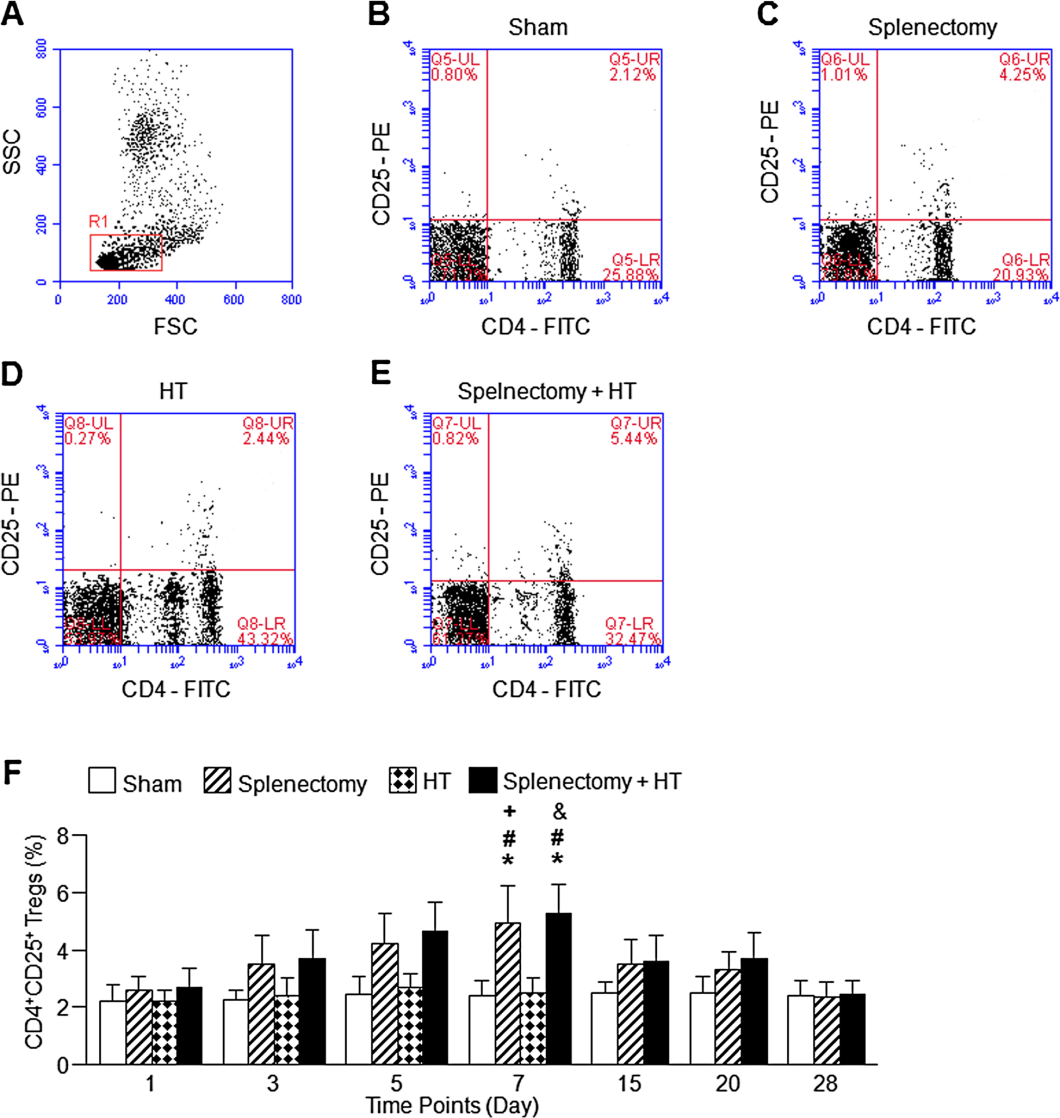
**Analysis of the level of CD4**^**+**^**CD25**^**+ **^**Tregs by flow cytometry.** Representative flow cytometric plots: **A**, the dot plot shows the typical location of lymphocyte population and its gate (R1); **B**-**E**, the quandary plots demonstrates the level of CD4^+^CD25^+^ Tregs at day 5 after transplantation of all experimental groups; **F**, summarized data were expressed as mean ± SEM, n = 5-15/group, *p < 0.05, *vs.* sham; ^#^ p < 0.05, *vs.* HT; ^+^ p < 0.05, *vs.* splenectomy at day 1 or day 28; ^&^ p < 0.05, *vs.* splenectomy + HT at day 1 or day 28.

The CD4^+^CD25^+^ Tregs in the PBMCs were determined by using the flow cytometry method. In the forward and side scatter plots, the typical lymphocyte population identified on basis of size and granularity was presented and a gate (R1) was set (Figure 3A). Representative flow cytometric panels showed the percentage of CD4^+^CD25^+^ Tregs within the gate (R1) in all experimental groups (Figure 3B-E) at day 5 after transplantation. We observed that there was a higher percentage of CD4^+^CD25^+^ Tregs (present in the upper right of the quarternary plot) in splenectomy and splenectomy + HT groups at day 5. Flow cytometric analysis revealed that the percentage of CD4^+^CD25^+^ Tregs was increased on day 3, 5, and 7 after splenectomy surgery (day 7: 2.4 ± 0.5%, 4.9 ± 1.3% and 5.3 ± 1.0% for sham, splenectomy and splenectomy + HT, respectively; n = 15/group, sham *vs.* splenectomy or splenectomy + HT, p < 0.05). Then, the level of CD4^+^CD25^+^ Tregs in recipient rats was gradually decreased to baseline level on day 28 (2.4 ± 0.5%, 2.4 ± 0.5% and 2.5 ± 0.5% for sham, splenectomy and splenectomy + HT, respectively; n = 5/group, sham *vs.* splenectomy or splenectomy + HT, p > 0.05). There were neither significant differences in the levels of CD4^+^CD25^+^ Tregs between sham and HT groups, nor between splenectomy and splenectomy + HT groups, indicating heart transplantation alone had no effect on the CD4^+^CD25^+^ Tregs level. These data were summarized in Figure 3F.

### The lymphocyte apoptotic rate was increased in splenectomized rats

**Figure 4:**
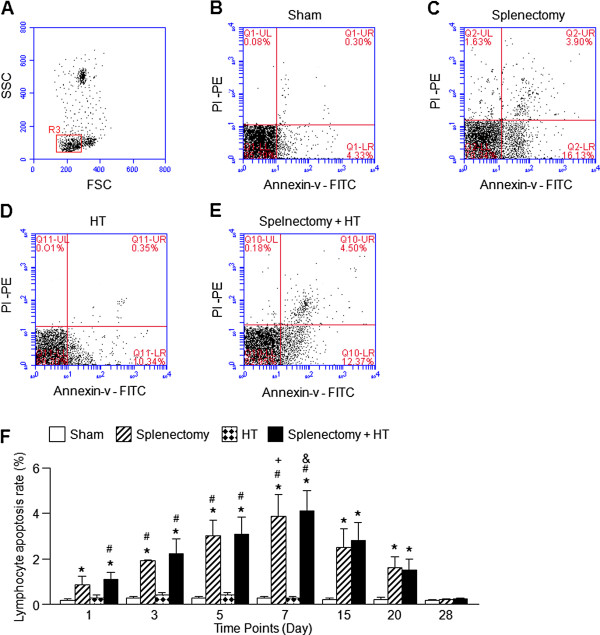
**Analysis of the lymphocyte apoptotic rate by flow cytometry.** Representative flow cytometric plots: **A**, the dot plot shows the typical location of lymphocyte population and its gate (R3); **B**-**E**, the quandary plots demonstrates the percentage of lymphocyte apoptosis at day 7 after transplantation of all experimental groups; **F**, summarized data were expressed as mean ± SEM, n = 5–15/group, *p < 0.05, *vs.* sham; ^#^ p < 0.05, *vs.* HT; ^+^ p < 0.05, *vs.* splenectomy at day 1 or day 3 or day 28; ^&^ p < 0.05, *vs.* splenectomy + HT at day 1 or day 3 or day 28.

In all experimental groups, the apoptotic rate in lymphocytes was analyzed by the flow cytometry method. In the forward and side scatter plots, the typical lymphocyte population identified on basis of size and granularity was presented and a gate (R3) was set (Figure 4A). Representative flow cytometric panels demonstrated the rate of lymphocyte apoptosis within the gate (R3) in all experimental groups (Figure 4B - E) at day 7 after heart transplantation. We found that there was a higher rate of lymphocyte apoptosis (present in the upper right of the quarternary plot) in splenectomy and splenectomy + HT groups at day 7. Flow cytometric analysis revealed that the percentage of annexin-v^+^ PI^+^ expression cells was increased on day 3, 5, and 7 after the splenectomy surgery (day 7: 0.3 ± 0.05%, 3.9 ± 0.9% and 4.1 ± 0.9% for sham, splenectomy and splenectomy + HT, respectively; n = 15/group, sham *vs.* splenectomy or splenectomy + HT, p < 0.05). Then, the lymphocyte apoptotic rate was gradually decreased to baseline level in recipient rats on day 28 (0.2 ± 0.01%, 0.2 ± 0.05% and 0.3 ± 0.01% for sham, splenectomy and splenectomy + HT, respectively; n = 5/group, sham *vs.* splenectomy or splenectomy + HT, p > 0.05). There were neither significant differences in the percentages of annexin-v^+^ PI^+^ cells between sham and HT groups, nor between splenectomy and splenectomy + HT groups, indicating heart transplantation alone had no effect on lymphocyte apoptosis. These data were summarized in Figure 4F.

### Pathological changes of transplanted hearts

**Figure 5:**
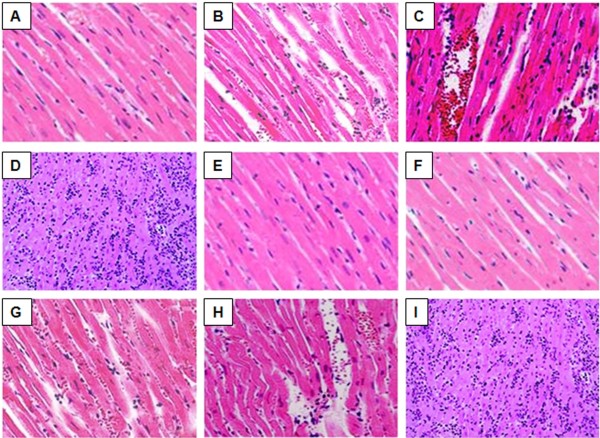
**Histological assessment of transplanted hearts.** Representative images of H&E staining (all magnifications: 20x): **A**, a heart section from a donor rat showing the normal structure of cardiac myofibers; **B**-**D**, heart sections collected from HT group at different time point; **B**, interstitial edema and inflammatory cell infiltration were observed on day 3 after transplantation; **C**, cardiomyocyte hemorrhage and an increasing number of inflammatory cell infiltrations were found on day 5 after transplantation; D, diffuse inflammatory infiltrate, myocardial cell necrosis, and destruction of myofibers were observed on day 7 after transplantation; **E**-**I**, heart sections collected from splenectomy + HT group at different time point; **E**-**F**, no pathology changes could be observed on days 3 and 5 after transplantation; **G**, mild interstitial edema and cellular infiltration was seen in the myocardium on day 7 after transplantation; **H**, cardiomyocyte interstitial edema and an increasing inflammatory cell infiltration could be observed on day 15 after transplantation; **I**, myocardial cell necrosis and destruction of myofibers were observed on day 28 after transplantation.

The histopathology of heart allografts were assessed at different time points (day 3, 5, 7, 15, and 28) in HT and splenectomy + HT groups. Figure 5A shows the histology of a normal rat heart. At day 3, the transplanted hearts from HT group (Figure 5B) had interstitial edema and inflammatory cell infiltration, which were absent in the allograft hearts from splenectomy + HT group (Figure 5E). At day 5, cardiomyocyte hemorrhage and an increasing number of inflammatory cell infiltrations were found in the transplanted hearts in HT group (Figure 5C). No pathologic change could be identified in the transplanted hearts from splenectomy + HT group (Figure 5F). At day 7, indications of severe rejections such as diffuse inflammatory infiltrate, myocardial cell necrosis, and destruction of myofibers appeared in the transplanted hearts from HT (Figure 5D), while there was mild cell infiltration in the myocardium of the allograft hearts from splenectomy + HT group (Figure 5G). At day 15, interstitial edema and increased inflammatory cell infiltration were observed in the allograft hearts collected from splenectomy + HT group (Figure 5H). At day 28, the transplanted hearts collected from splenectomy + HT group were soft and partially gray-white with focal edema in the subepicardium. Upon examination with a microscope, severe rejection of the transplanted hearts showed myocardial cell necrosis and destruction of myofibers (Figure 5I).

## Discussion

The major finding of the present study is that splenectomy can suppress the development of pathology and prolong the mean survival time of the cardiac allograft. Our data suggested that splenectomy plays a critical role in the development of immune tolerance in heart transplantation by increasing the level of CD_4_^+^CD_25_^+^ Tregs and promoting the apoptosis of lymphocytes.

Graft rejection by the immune system is a major cause of transplant failure. As we know, the largest lymphatic organ, the spleen, provides an immune microenvironment which accepts antigen stimulation and causes an immune response. Therefore, splenectomy is able to eliminate a primary immune response to an allograft. An earlier study [8] has reported that splenectomy is beneficial for xenograft survival by blocking the humoral antibody response.

The CD4^+^CD25^+^ Tregs, which constitute 5 - 10% of peripheral CD4^+^ T cells in mice and humans, are recognized as a major subset of immune cells possessing potent suppressive properties [14,15]. A study on a thymectomised mouse model showed that CD4^+^CD25^+^ Tregs can prevent autoimmunity and allergy [16]. Moreover, several transplantation studies [7,8] have demonstrated that CD_4_^+^CD_25_^+^ Tregs can effectively prevent transplantation rejection by blocking the initiation of the immune response against the graft and actively participating in the regulation of the immune tolerance. In consistence with those reports, we found that the level of CD_4_^+^CD_25_^+^ Tregs was significantly elevated within the first 7 days after the splenectomy surgery and gradually decreased to the baseline level, and the histopathologic change of transplanted hearts was milder and the mean survival time of transplanted hearts was longer in splenectomized rats. The possible explanation of this phenomenon was that accumulation of CD_4_^+^CD_25_^+^ Tregs inhibited the activation of naive T cells of the recipient, creating a permissive environment for graft acceptance. That is why CD_4_^+^CD_25_^+^ Tregs were increased in the early days (within 7 days) after the splenectomy. However, due to some of the CD_4_^+^CD_25_^+^ Tregs being generated from the CD_4_^+^CD_25_ - population in the adoptive system in the spleen [17,18], the CD_4_^+^CD_25_ - population was decreased after the splenectomy, which could explain why the CD_4_^+^CD_25_^+^ Tregs were gradually back to the normal level later.

The mechanisms responsible for CD_4_^+^CD_25_^+^ Tregs immunity suppression are not fully understood, and many of these mechanisms remain controversial [6,19]. Some observations have shown that one of the immunesuppression mechanisms was CD_4_^+^CD_25_^+^ Tregs induced apoptosis of the immune cells [20-24]. Since lymphocyte apoptosis is an important immunoregulatory mechanism for maintaining homeostasis in the immune system *in vivo*[25-27], which could be an effective strategy to reduce transplantation rejection. Sharland *et al.*[28] reported that tolerant rats had higher numbers of apoptotic activated T cells in a liver transplantation model. In this study, we found that splenectomy regulated the immune homeostasis *in vivo* by increasing the rate of lymphocyte apoptosis. The flow cytometric results demonstrated that the rate of lymphocyte apoptosis was significantly increased in the splenectomized rats within the first 7 days after the splenectomy surgery and gradually decreased to baseline level. At the same time, this increased apoptosis tendency paralleled the raised level of CD_4_^+^CD_25_^+^ Tregs. These data are in agreement with the previous findings [21-23,28].

In this study, we first reported that splenectomy prolonged the survival time of cardiac allograft by enhancing the immune tolerance via raising the level of CD_4_^+^CD_25_^+^ Tregs and increasing the apoptotic rate of lymphocyte. The spleen has several unique functions in the immune system concerning not only the CD_4_^+^CD_25_^+^ Tregs, but also various kinds of cytokines such as interleukin-10 and transforming growth factor (TGF)-β. Our future study on these cytokines will better ascertain the immunomodulatory role of splenectomy in the cardiac allograft.

## Conclusions

Our data indicate that splenectomy inhibits the development of pathology and prolongs the survival time of cardiac allograft. The responsible mechanism is associated with the induction of immune tolerance via elevating CD_4_^+^CD_25_^+^ Tregs and increasing lymphocyte apoptosis.

## Competing interest

There is no competing interest.

## Authors’ contributions

JZ, YC and ZY participated in research design; JZ, WZ, PL and RM performed the experiments; JZ, JW, SC, YC and ZY participated in the writing of the manuscript; JZ, JW, SC, CZ, YC and ZY participated in data analysis. All authors read and approved the final manuscript.
